# PGS/HAp Microporous Composite Scaffold Obtained in the TIPS-TCL-SL Method: An Innovation for Bone Tissue Engineering

**DOI:** 10.3390/ijms22168587

**Published:** 2021-08-10

**Authors:** Paweł Piszko, Marcin Włodarczyk, Sonia Zielińska, Małgorzata Gazińska, Przemysław Płociński, Karolina Rudnicka, Aleksandra Szwed, Agnieszka Krupa, Michał Grzymajło, Agnieszka Sobczak-Kupiec, Dagmara Słota, Magdalena Kobielarz, Magdalena Wojtków, Konrad Szustakiewicz

**Affiliations:** 1 Department of Polymer Engineering and Technology, Faculty of Chemistry, Wrocław University of Science and Technology (WUST), Wyb. Wyspiańskiego 27, 50-370 Wrocław, Poland; sonia.zielinska@pwr.edu.pl (S.Z.); malgorzata.gazinska@pwr.edu.pl (M.G.); michal.grzymajlo@pwr.edu.pl (M.G.); 2 Department of Immunology and Infectious Biology, Faculty of Biology and Environmental Protection, University of Łódź, Banacha 12-16, 90-237 Łódź, Poland; marcin.wlodarczyk@biol.uni.lodz.pl (M.W.); przemyslaw.plocinski@biol.uni.lodz.pl (P.P.); karolina.rudnicka@biol.uni.lodz.pl (K.R.); aleksandra.szwed@biol.uni.lodz.pl (A.S.); agnieszka.krupa@biol.uni.lodz.pl (A.K.); 3 Institute of Materials Science, Faculty of Materials Science and Physics, Cracow University of Technology, 37 Jana Pawła II Av., 31-864 Krakow, Poland; agnieszka.sobczak-kupiec@pk.edu.pl (A.S.-K.); dagmara.slota@doktorant.pk.edu.pl (D.S.); 4 Department of Mechanics, Materials and Biomedical Engineering, Faculty of Mechanical Engineering, Wroclaw University of Science and Technology (WUST), Wyb. Wyspiańskiego 27, 50-370 Wrocław, Poland; magdalena.kobielarz@pwr.edu.pl (M.K.); magdalena.wojtkow@pwr.edu.pl (M.W.)

**Keywords:** poly(glycerol sebacate), thermal induced phase separation, osteoblasts, osteoconductive, osteoclastogenic cytokines, in vivo biocompatibility, scaffolds

## Abstract

In this research, we synthesize and characterize poly(glycerol sebacate) pre-polymer (pPGS) (^1^H NMR, FTiR, GPC, and TGA). Nano-hydroxyapatite (HAp) is synthesized using the wet precipitation method. Next, the materials are used to prepare a PGS-based composite with a 25 wt.% addition of HAp. Microporous composites are formed by means of thermally induced phase separation (TIPS) followed by thermal cross-linking (TCL) and salt leaching (SL). The manufactured microporous materials (PGS and PGS/HAp) are then subjected to imaging by means of SEM and µCT for the porous structure characterization. DSC, TGA, and water contact angle measurements are used for further evaluation of the materials. To assess the cytocompatibility and biological potential of PGS-based composites, preosteoblasts and differentiated hFOB 1.19 osteoblasts are employed as in vitro models. Apart from the cytocompatibility, the scaffolds supported cell adhesion and were readily populated by the hFOB1.19 preosteoblasts. HAp-facilitated scaffolds displayed osteoconductive properties, supporting the terminal differentiation of osteoblasts as indicated by the production of alkaline phosphatase, osteocalcin and osteopontin. Notably, the PGS/HAp scaffolds induced the production of significant amounts of osteoclastogenic cytokines: IL-1β, IL-6 and TNF-α, which induced scaffold remodeling and promoted the reconstruction of bone tissue. Initial biocompatibility tests showed no signs of adverse effects of PGS-based scaffolds toward adult BALB/c mice.

## 1. Introduction

Due to their potential applications in regenerative medicine, biodegradable polymeric materials are becoming increasingly popular among researchers from around the world. There are numerous scientific articles on, e.g., polyester synthesis [1,2], physical modification [3,4] or formation [5,6,7]. The most popular polyester with a potential in regenerative medicine is PLLA [8]. Researchers have focused on PLLA’s biodegradability and biocompatibility as well as its good mechanical and thermal properties [9]. Other biodegradable polyesters, including polycaprolactone [10,11] or poly(l-lactide-co-glycolide) [12,13], are very popular due to their potential applicability.

One of the relatively new polyesters that is lately becoming increasingly sought-after is poly(glycerol sebacate) (PGS) [14,15]. PGS was first obtained and described in 2002 as a new biocompatible elastomer [16]. In this research, the polymer was synthesized in the equimolar ratio of monomers in inert gas (argon) mTorr at 120 °C for 24 h. Next, the pressure was reduced to 40 mTorr over the course of 5 h, and the reaction mixture was kept at 120 °C for another 48 h. Variations of the above synthetic approach were used in a handful of other studies [17,18,19]. Other polymerization methods, including enzymatic [20,21] and microwave synthesis [22] and photopolymerization [23] have been reported. In terms of its properties, PGS is very attractive for biological applications. Hence, the monomers for PGS synthesis are of endogenous origin (i.e., glycerol and sebacic acid) [16,24,25], and the polymer and its composites exhibit biocompatible properties [26,27]. Moreover, PGS is considered biodegradable [18,28,29]. All these properties are desirable in tissue engineering. Furthermore, the mechanical properties of poly(glycerol sebacate) resemble these of soft tissues [28,29], and PSG is tougher than the commonly encountered crosslinked biomaterials [16]. Finally, PGS is considered an elastomeric material [19], which is another desirable property for biological applications and the formation of composites.

One of the most important techniques for producing a cellular scaffold for tissue engineering is Thermally Induced Phase Separation (TIPS). The TIPS process was patented by A. Castro in 1981 [30] and initially introduced as a process of manufacturing microporous membranes [31]. However, this process was successfully adapted to manufacture not only flat membranes but also 3-dimensional scaffolds with high porosity [32]. The operating principle of TIPS is based on de-mixing (phase separation) of the polymer solution followed by freeze-drying in order to remove the solvent [33]. The process occurs in the presence of the porogen, which can be leached after the process to obtain a scaffold with interconnected pore structures of desired size. The most commonly encountered porogens are sodium chloride, saccharose and glucose [33,34,35] with a uniform grain-size; the fractions are suitably sieved. In order to perform TIPS successfully, the solvent needs to be thermolabile to be subjected to freeze drying. Moreover, it must dissolve the polymer matrix, but must not affect the blowing agent; e.g., 1,4-dioxane will dissolve poly-L-lactide but will not dissolve salt (porogen) [7]. Thus far, there are several reported potential applications for PGS in medicine, including vascular grafts [36], drug carrier [37], tissue engineering [38]—cartilage regeneration [39] and cardiac tissue engineering [40,41].

In this article, a solventless, equimolar polycondensation synthesis was applied in atmospheric pressure and elevated temperature to obtain pPGS. A 3D porous scaffold based on the PGS/HAp material and using the TIPS-TCL-SL method was obtained. This composite can be used as an innovative scaffold for bone tissue regeneration. 

## 2. Results and Discussion

### 2.1. pPGS Chemical and Structural Analysis

As shown in Figure S1, the ATR-FTIR spectra of the pPGS prepolymer, PGS and PGS/HAp composites revealed the presence of bands centered around: 3400 cm^−1^ (OH stretching); 2927 cm^−1^ (CH2 antisymmetric stretching); 2854 cm^−1^ (CH_2_ symmetric stretching); 1733 cm^−1^ (C=O stretching); 1456 cm^−1^ (CH_2_ scissoring); 1165, 1197 and 1047 cm^−1^ (C-O stretching in 3°, 2° and 1° alcohol); 923 cm^−1^ (OH deformation); and 723 cm^−1^ ((CH_2_)_n_ rocking) [42,43,44,45,46]. The presence of new ester bonds in the pre-polymer and the PGS crosslinked network was confirmed by the shift of the peak located around 1730 cm^−1^ from lower values in sebacic acid (typical for free acid groups) toward higher values (typical for carbonyl groups in esters). In the PGS/HAp composite spectrum, there is a strong band related to the PO_4_ vibrations, related to phosphates in hydroxyapatite particles [47,48].

The ^1^H NMR spectrum (Figure S2) is a valuable source of information for the details of the PGS polymer structure. The pPGS pre-polymer is composed of three possible glyceridic units—linear L12 and L13 type diglyceridic repetitive units, D triglyceridic units and terminal monoglyceridic units (T1 and T2). In the chemical shift range from 1.3 ppm to 2.3 ppm, there are signals related to sebacic acid or sebacate units. In the region from 3.4 ppm to 4.5 ppm, there are overlapping signals from glycerol and glyceridic units, and, in the range from 4.8 ppm to 5.3 ppm, we can find signals from units with esterified secondary hydroxyl groups (L12, D and T2) [21].

Previous reports on the 1D and 2D ^1^H NMR spectra of pPGS published elsewhere proved that the signals related to the methine protons of T1, T2, L12 and D glyceridic units are free of overlapping [21]. Therefore, the areas of these signals, denoted by 4 (3.80 ppm), 5 (4.84 ppm), 2 (5.04 ppm) and 3 (5.24 ppm) in Figure S2 and Table S1, were used to calculate the molar fractions of each glycideric unit (x_i_) in the polycondensation product. For normalization purposes, the area of signal a was used [21].

The pPGS molecular weight distribution curve (see Figure S3) presents the polydispersity of pPGS. We outline five significant peaks. The GPC results indicated that the broadest peak (66.84% peak area) of the synthesized pPGS had a number-average molecular weight of M_n_ = 6362 Da, weight-average molecular weight of M_w_ = 11,867 Da, viscosity-average molecular weight of M_z_ = 26,445 Da and a polydispersity index (PDI) on the level of 1.865.

### 2.2. PGS/HAp Scaffold Structure and Morphology

**Figure 1:**
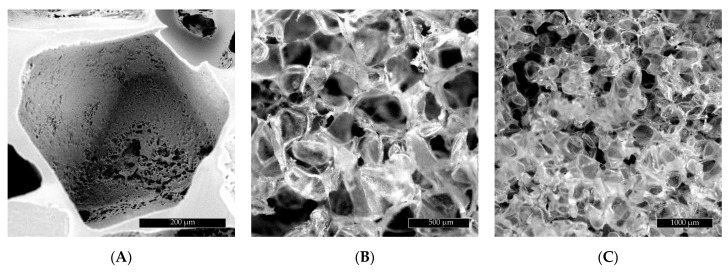
(**A**) SEM images of the PGS scaffold pores. (**B**) Optical image of the PGS scaffold. (**C**) Optical image of the PGS scaffold.

**Figure 2:**
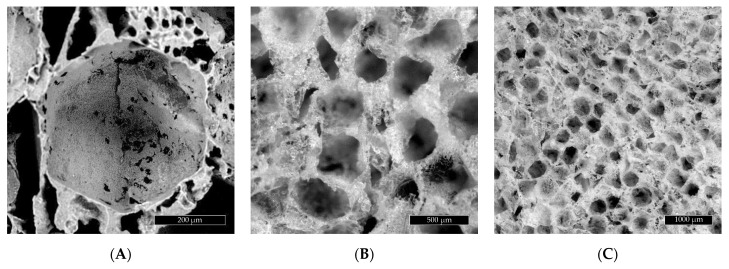
(**A**) SEM images of the PGS/HAp scaffold pores. (**B**) Optical image of the PGS/HAp scaffold. (**C**) Optical image of the PGS/HAp scaffold.

The scaffold structural images obtained by means of SEM and stereoscopic microscopy are shown in Figure 1 (PGS) and Figure 2 (PGS/HAp). A morphological difference between the wall structure can be observed in the images of both specimens. The PGS scaffold walls appear solid, while the addition of hydroxyapatite conceives a shredded appearance of the sample with more empty areas between the pores. The diameter measurement of the pores in Figure 1 and Figure 2 shows an average diameter of 434.1 µm for the PGS scaffold and 483.7 µm for PGS/HAp. These results fit in size the range of the NaCl grains (400–500 µm) used as a porogen in the scaffold manufacturing process.

**Figure 3:**
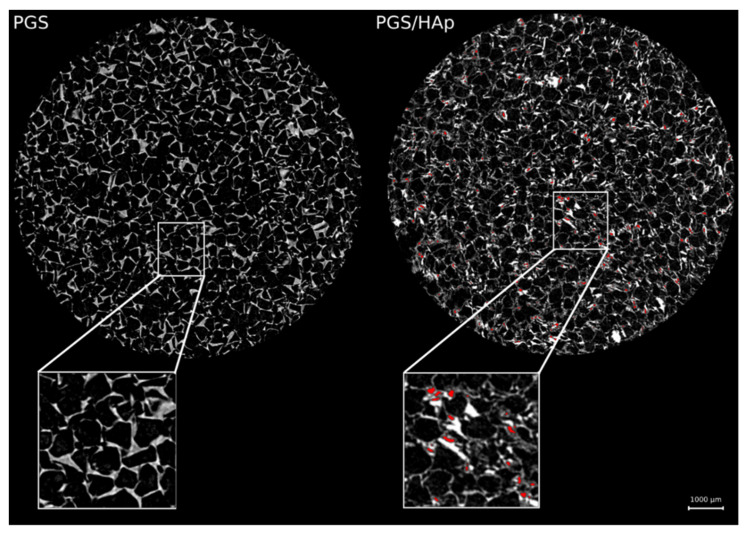
Representative cross-sections (µCT) of foams showing the differences in pore shape. The micropores occurring within the structure of walls are marked in red.

The internal microarchitecture diversity of PGS and PGS foams with hydroxyapatite was defined, i.e., the shape and size of pores and structural arrangement of the materials (Figure 3). Numerous delamination and discontinuities of the structure were visible in the PGS/HAp foams and also noted in SEM images (Figure 2).

**Figure 4:**
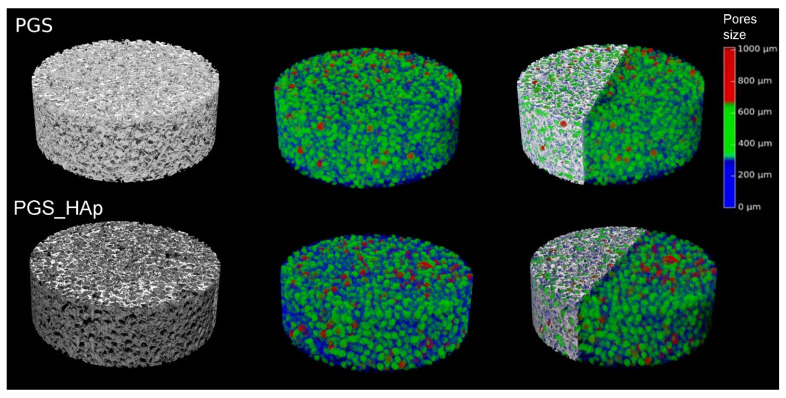
Reconstruction of foam structures and color-coded pore size representation. Grey colors represent the scaffold wall structure, while blue, green and red colors represent pores with different diameters. Diameters from the range 0–300 µm are blue, 300–650 µm—green and more than 650 µm—red.

A quantitative analysis of the foams’ internal structure was carried out, including porosity, pore diameter and sphericity. The reconstruction of both foam types and the obtained total porosity (color-coded in terms of size) is shown in Figure 4.

The total porosity (Po(tot)) of the PGS foam was 78.0% relative to 69.8% for the PGS/HAp sample. Mostly it was the open porosity (the closed porosity was extremely low, i.e., less than 0.001% and 0.02% for the PGS and PSG/HAp foams, respectively).

**Figure 5:**
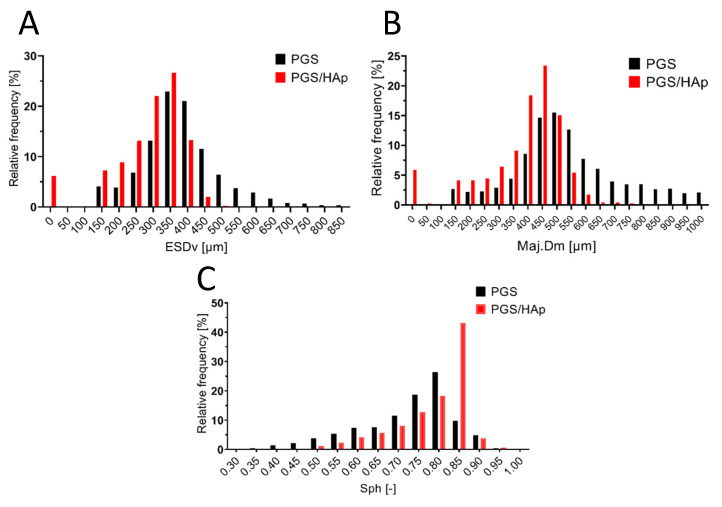
Pore diameter described as (**A**) a volume-equivalent sphere diameter (ESDv), (**B**) major diameter (Maj.Dm), (**C**) sphericity (Sph). Values on the X axis are presented as a bin center.

The volume of the Po.V pores was 216.01 mm^3^ and 193.39 mm^3^ in the PGS and PGS/HAp samples, respectively. The histogram obtained for the mean ESDv showed that the PGS foam had pores with a diameter ranging from 150 to 850 µm, while, in the case of PSG/HAp, the largest pores had a diameter of 450 µm (Figure 5A). However, in the PGS/HAp foam, small pores localized within the foam wall structure, with a diameter of around 50 µm (or below 75 µm), were observed (Figure 2), and the pore diameter histograms showed their presence at a level of 6% (Figure 5A,B). The mean pore size (ESDv) in the PSG/HAp sample was 286 ± 103 µm, while the major diameter (Maj.Dm) equaled 385 ± 147 µm. When the micropores observed within the walls of this sample were excluded from analysis, this structure was characterized with ESDv equal to 304 ± 77 µm and Maj.Dm equal 410 ± 115 µm. For comparison, in the PSG sample, ESDv values around 383 ± 120 µm and Maj.Dm around 596 ± 271 µm were observed. The pore sphericity (Sph, equal 1 for ideally spherical porosity) analyzed for the PGS and PGS/HAp foams equaled 0.72 ± 0.12 [-] and 0.81 ± 0.05 [-] (*p* < 0.0001, Mann–Whitney test). The PGS/HAp foam pores were more homogeneous, showing a similar, more spherical shape compared to PGS. For this type of foam, the Sph ranged from 0.35 to 0.90 [-] (Figure 5C). All the parameters are listed in Table 1.

### 2.3. Scaffolds Contact Angle

**Figure 6:**
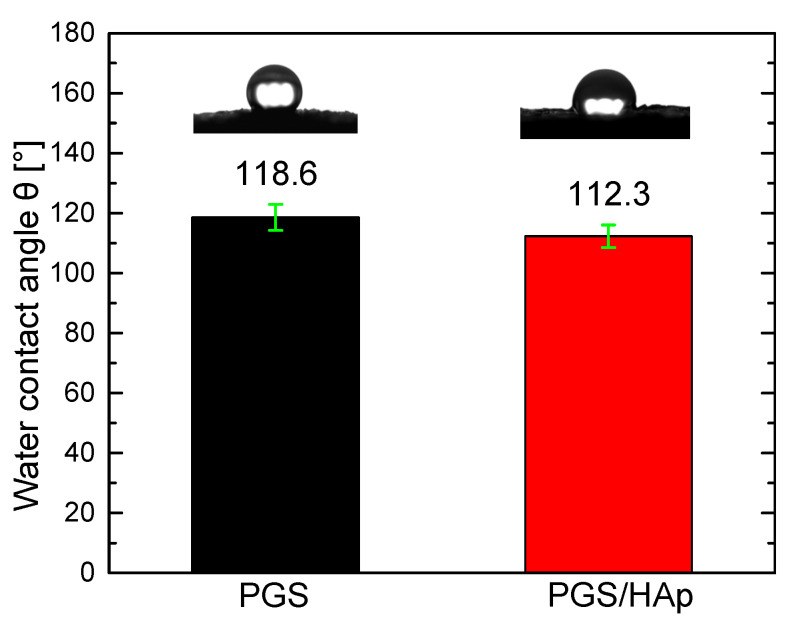
Water contact angle measurement (mean value ± SD) for the PGS and PGS/HAp scaffolds.

On average, the water contact angle measurement indicated 118.6° ± 4.3 for the PGS scaffolds and 112.3° ± 3.8 for the PGS/HAp scaffolds (Figure 6). The results indicate an increase in hydrophilicity after the addition of Hap, which is consistent with a previously reported study [49]. Multiple articles reported the hydrophilic character of bulk PGS ranging from 32° [16] to around 70–80° [49,50,51]. The porous morphology highly affects the contact angle on the researched scaffolds. Similar water contact angles were achieved (105.23–111.90°) for a study in which porous PGS scaffolds manufactured by the salt-leaching method were investigated [52].

### 2.4. Scaffolds Thermal Analysis

**Figure 7:**
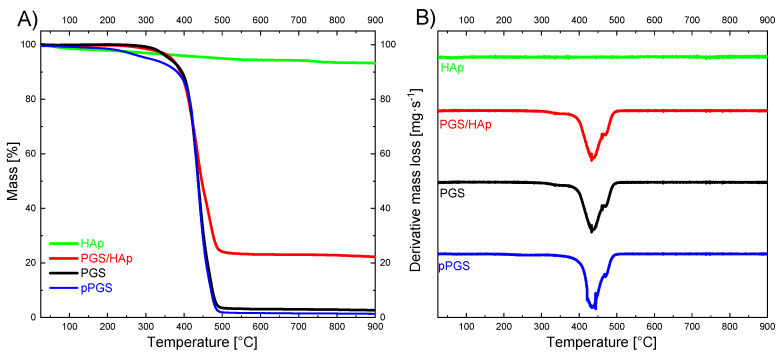
(**A**) Thermogravimetric curves of pPGS and HAp as well as PGS/HAp 75/25 and the PGS porous scaffolds. (**B**) First derivative of the TGA curves of pPGS and HAp as well as PGS/HAp 75/25 and the PGS porous scaffolds.

Based on the TGA analysis degradation mechanism, the thermal stability of the pre-polymer pPGS and the foams was determined and the hydroxyapatite content in composite foam PGS/HAp 75/25 was estimated. The TGA curves and the first derivative of the mass with respect to time (dm/dt = f(t)) (dTGA curves) are shown in Figure 7. Thermal stability values estimated as temperature corresponding to 5 wt.% of mass loss (T_-5%_) are collected in Table 1. The T_-5%_ of crosslinked PGS is about 55 °C higher than that of the prepolymer pPGS. Moreover, due to the rgw presence of HAp, the thermal stability of composite foams was additionally improved by about 7 °C (Table 2).

The HAp content in the composite foam was calculated from the residue at 700 °C taking into account the 5.79% mass loss of neat hydroxyapatite and assuming that the carbon black residue at 700 °C from the degradation of crosslinked PGS stands at 1.53% of PGS content as estimated for the neat PGS100 foam. The calculated HAp content of 23.18 wt.% indicates a good agreement between the estimated and the processing filler contents.

**Figure 8:**
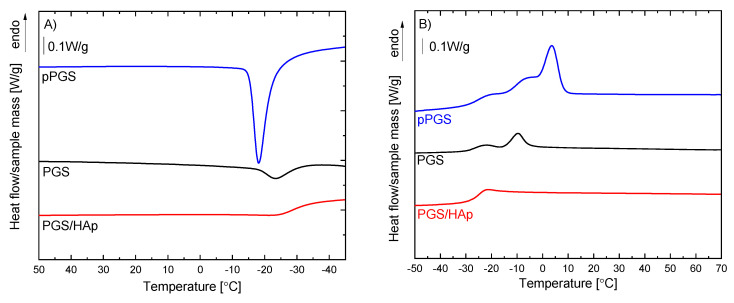
The cooling (**A**) and second heating (**B**) DSC curves of prepolymer pPGS and porous scaffolds PGS and PGA/HAp.

In order to compare the thermal properties of the synthesized pre-polymer pPGS and the porous PGS and PGS/HAp scaffolds, the differential scanning calorimetry measurements were performed. It is known that PGS exhibits a semi-crystalline character [22,53]. The thermal properties were evaluated from the cooling and second heating DSC curves, because the first heating curves are influenced by the different thermal histories of the synthesized pPGS and the PGS and PGS/HAp foams formed by thermally induced phase separation followed by thermal crosslinking and salt leaching. The cooling and second heating DSC curves are presented in Figure 8, and the estimated thermal parameters are collected in Table 3. As indicated by the exothermic peak on the cooling curves of pPGS and PGS, the pre-polymer and polymeric foam crystallized from the melt, whereas the composite foams did not exhibit melt crystallization on cooling with the heating rate used (10 °C/min). Melt crystallization of the composite foam was partially restricted due to the crosslinking of PGS, when comparing between the crystallization behavior of pPGS and PGS. However, the lack of melt crystallization of the composite foam is mainly caused by the high content of HAp particles. Thus, for the composite foam on the cooling curve, only glass transition was visible with the glass transition temperature (T_g_) at −28.6 °C. Due to crosslinking, the melt crystallization of PGS shifted to lower temperatures compared to pPGS. The peak temperature of the melt crystallization (T_c_) of PGS was lower by about 5.8 °C compared with that of pPGS, and the enthalpy of melt crystallization (ΔH_c_) of PGS was lower than that of the pre-polymer. The Tg of pPGS and PGS cannot be estimated reliably, because of the overlapping of the melt crystallization exothermic reaction on the glass transition range. However, the presence of a glass transition clearly indicates an increase of the baseline level visible at temperatures below melting, recorded for pPGS. The Tg values evaluated from the second heating DSC curves were −25.1 °C, −25.8 °C and −24.9 °C for pPGS, PGS and PGS/HAp, respectively. Above the glass transition, a melting peak was recorded for pPGS and PGS. The melting peak of PGS was located at 3.3 °C. On the leading edge of the melting peak of pPGS, a second maximum was located, and the main melting peak was at −9.4 °C. The melting enthalpy of pPGS was higher than of PGS, indicating a higher crystallinity degree of pPGS. Concluding, the DSC experiments revealed that the composite foam was amorphous, whereas pPGS and PGS were semicrystalline with a lower crystallinity degree of the PGS foam.

### 2.5. Cytocompatibility and Osteoconductivity

#### 2.5.1. Colonization of Scaffolds

**Figure 9:**
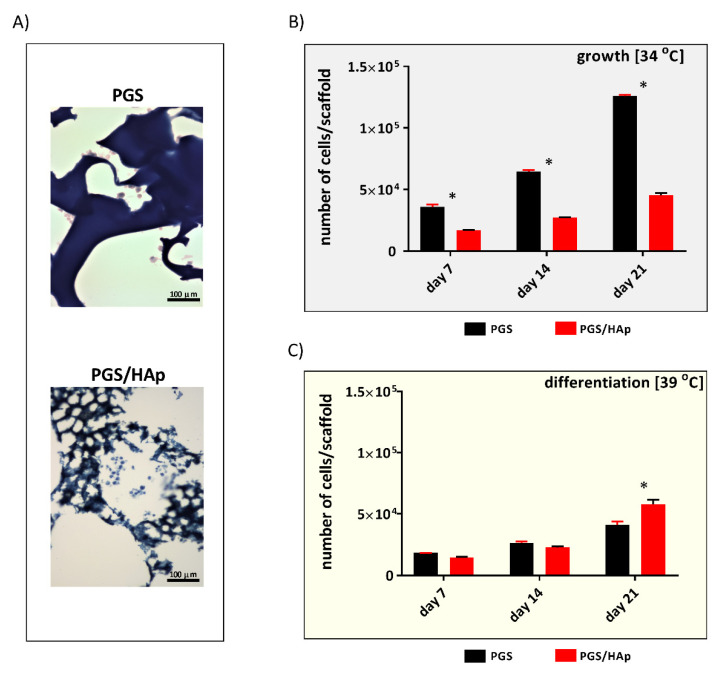
Optical microscopic images of May–Grunwald–Giemsa stained sections of the PGS and PGS/HAp scaffolds populated by hFOB 1.19 pre-osteoblasts in 7-day cell cultures (**A**). The nuclei of the cells appeared deep purple with light purple surroundings. The proliferation of human osteoblasts in the PGS and PGS/HAp scaffolds (**B**) under conditions permitting proliferation (34 °C) and (**C**) in the osteogenic conditions (39 °C) followed for up to 21 days of culture. The results are shown as the mean values ± SD. * *p* < 0.05 between the PGS and PGS/HAp composites, with the Mann–Whitney U test used to assess the statistical significance for the observed changes.

Due to the intrinsic broad range autofluorescence of the PGS/HAp composites, May–Grunwald–Giemsa staining was used to help monitor the behavior and visualize hFOB 1.19 cells cultured on the HAp scaffolds instead of confocal microscopy. As evidenced by staining, the cells successfully colonized the scaffold, attached and were able to grow on both PGS and PGS/HAp after 7 days of incubation (Figure 9A).

#### 2.5.2. hFOB 1.19 Proliferation

The hFOB1.19 human fetal osteoblast is a temperature sensitive, SV40-transfected cell line, which resembles preosteoblasts when grown at conditions permitting cell proliferation (34 °C). The cells stopped dividing and began to differentiate into mature osteoblasts at a non-permissive temperature of 39 °C. Following the initial stages of invasion and settlement of the cells populating the scaffolds, the cell proliferation patterns were recorded at 7, 14 and 21 days of culture with the use of the CyQUANT proliferation assay (Figure 9B,C).

It is noteworthy that the number of pre-osteoblasts cultured on both types of scaffolds continued to increase over time until day 21. However, under the permissive culturing conditions (34 °C), the number of osteoblasts detected on PGS scaffolds was significantly higher at all time points (3.5 × 10^4^ ± 2.0 × 10^3^, 6.4 × 10^4^ ± 1.2 × 10^3^, 1.2 × 10^5^ ± 1.0 × 10^3^, respectively) as compared to the number of hFOB 1.19 cells evaluated on PGS/HAp (1.6 × 10^4^ ± 3.3 × 10^2^, *p* = 0.002; 2.7 × 10^4^ ± 1.3 × 10^2^ ×, *p* = 0.002; 4.5 × 10^4^ ± 1.8 × 10^3^, *p* = 0.002; respectively). Number of cells cultured in osteogenic conditions (39 °C) revealed the expected reduction of the proliferation activity. In the case of differentiated hFOB1.19, there was a much more subtle increase in cell number, more pronounced on the PGS/HAp scaffolds on day 21, when compared to the cells grown on unmodified PGS foams (5.8 × 10^4^ ± 3.7 × 10^3^, 4.1 × 10^4^ ± 2.6 × 10^3^, *p* = 0.002, respectively). Considering the rather fragile nature of the osteoblastic hFOB1.19 cell line observed by us in the routine handling and based on our results, it can be assumed that the novel PGS/HAp scaffolds not only exhibit optimal cell biocompatibility in vitro but also support growth of differentiated osteoblasts in a long-term set-up. On the other hand, the addition of HAp slowed down the growth of hFOB 1.19 under conditions supporting cell proliferation, possibly signaling the cells to initiate the differentiation program rather than multiply.

#### 2.5.3. Osteogenic Differentiation of hFOB 1.19

**Figure 10:**
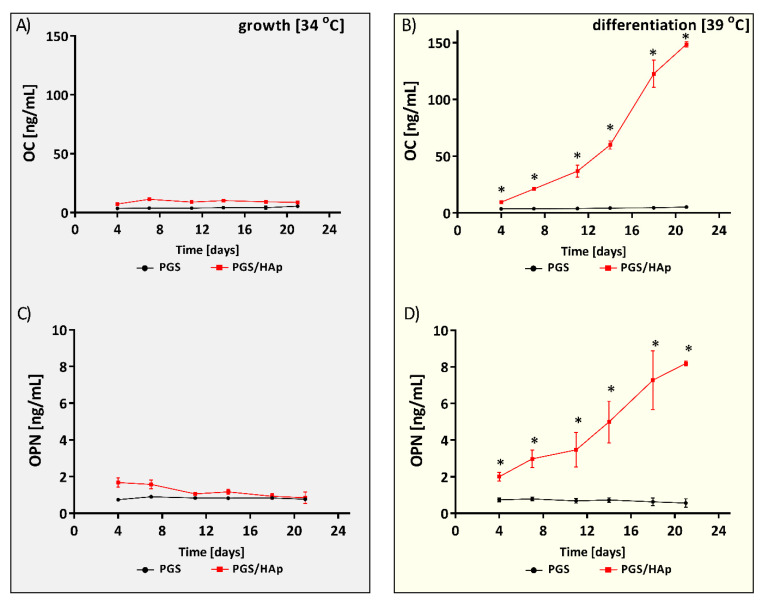
Analysis of the osteogenic markers osteocalcin (OC) and osteopontin (OPN) at conditions facilitating cell proliferation (34 °C) (**A**,**C**) or under osteoinductive conditions (39 °C) (**B**,**D**). The results represent the mean concentration ± SD. * *p* < 0.05 between the PGS and PGS/HAp composites, based on the Mann–Whitney U analysis.

The biomarkers of bone turnover are involved in the processes of bone metabolism and remodeling, including bone formation and bone resorption. Osteocalcin (OC), produced by osteoblasts, plays a role in metabolic regulation and bone mineralization, while osteopontin (OPN) is one of the major bone resorption biomarkers [54,55]. Osteogenic markers produced during the hFOB 1.19 differentiation, including OC and OPN, were detected in the supernatants collected on days 4, 7, 11, 14, 18 and 21 of osteoblast culture on the PGS and PGS/HAp scaffolds (Figure 10). Under culture conditions supporting cell proliferation (34 °C), the concentration of OC was similar at every time point, and its production was not affected by HAp. The level of OC produced by hFOB 1.19 on the PGS scaffold was 3.5 ± 1.7 ng/mL on day 4 and reached 5.4 ± 0.1 ng/mL on day 21. The levels of OC produced by the cells grown on the PGS/HAp composites were 7.2 ± 1.5 ng/mL on day 4 and reached 8.7 ± 1.3 ng/mL on day 21. Interestingly, when hFOB1.19 cells were cultured in an osteogenic environment (39 °C), the concentration of OC continued to accumulate on biomaterials enriched with HAp. Irrespective of the culturing temperature and the addition of osteogenic agents, the levels of OC remained unchanged throughout the experiment when cells were cultured in the absence of HAp. The levels of OC produced on the PGS/HAp composites were over one order of magnitude higher than those detected in cultures incubated in the absence of HAp, for which the OC production remained at the background level. The respective levels of OC produced in the presence of HAp compared to the OC production by osteoblasts cultured on unmodified PGS composites were 9.5 ± 2.5 ng/mL and 7.2 ± 1.5 ng/mL, *p* = 0.04 on day 4 and 148.5 ± 3.3 ng/mL and 8.7 ± 1.3 ng/mL, *p* = 0.01 on day 21.

The analysis of the OPN production revealed a similar tendency for OC. At 34 °C, the concentration of OPN remained low, at similar levels during the entire experiment, and was not related to the composition of scaffold. The level of OPN produced by hFOB 1.19 on the PGS scaffold was 0.7 ± 0.1 ng/mL on day 4 and remained unchanged at 0.7 ± 0.1 ng/mL on day 21. For the PGS/HAp composites, the concentration was 1.6 ± 0.6 ng/mL on day 4 and dropped down to 0.8 ± 0.4 ng/mL on day 21. Again, under osteoinductive conditions (39 °C), the concentration of OPN produced by hFOB 1.19 cultured on PGS/HAp scaffold was growing significantly and was higher as compared to the OPN production by osteoblasts propagated on the PGS composites: 2.0 ± 0.5 ng/mL and 1.6 ± 0.6 ng/mL, *p* = 0.04 on day 4 and 8.1 ± 0.1 ng/mL and 0.8 ± 0.4 ng/mL, *p* = 0.01 on day 21.

**Figure 11:**
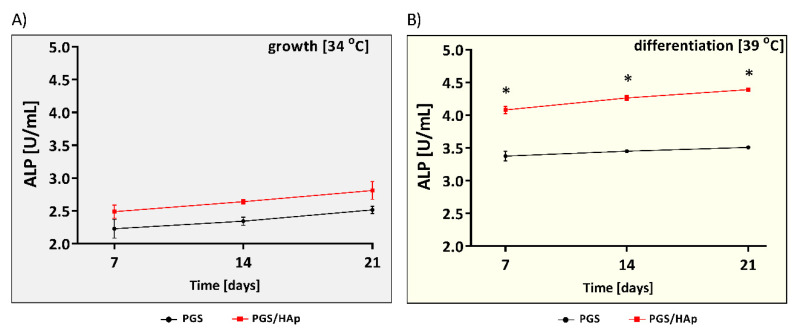
Alkaline phosphatase (ALP) activity in cell lysates obtained from hFOB1.19 cells cultured at 34 °C (**A**) or in the osteoinductive environment at 39 °C (**B**). The results represent the mean ALP activity ± SD. * *p* < 0.05 between the PGS and PGS/HAp composites, based on the results of the Mann–Whitney U test.

Alkaline phosphatase (ALP) reflects bone calcification and is necessary in the formation of hard tissue [56]. In the current study, we evaluated the ALP activity in cell lysates obtained from scaffolds colonized by hFOB 1.19 on day 7, 14 and 21 cultured at 34 or 39 °C (Figure 11). We noted no differences between the level of ALP produced by osteoblasts on the PGS and PGS/HAp scaffolds, when cells were cultured at 34 °C (2.2 ± 0.2 U/mL and 2.4 ± 0.1 U/mL on day 7; and 2.5 ± 0.1 U/mL and 2.8 ± 0.2 U/mL on day 21, respectively).

In contrast, in the osteoinductive environment, the lysates of hFOB 1.19 revealed an increased ALP activity on the PGS/HAp composites as compared to the lysates obtained from cells propagated on unmodified PGS scaffolds (4.1 ± 0.1 U/mL and 3.3 ± 0.1 U/mL, *p* = 0.02 (day 7); 4.2 ± 0.1 U/mL and 3.4 ± 0.1 U/mL, *p* = 0.02 (day 14); and 4.3 ± 0.1 U/mL and 3.5 ± 0.1 U/mL, *p* = 0.02 (day 21)).

#### 2.5.4. Production of Immunomodulatory Cytokines

**Figure 12:**
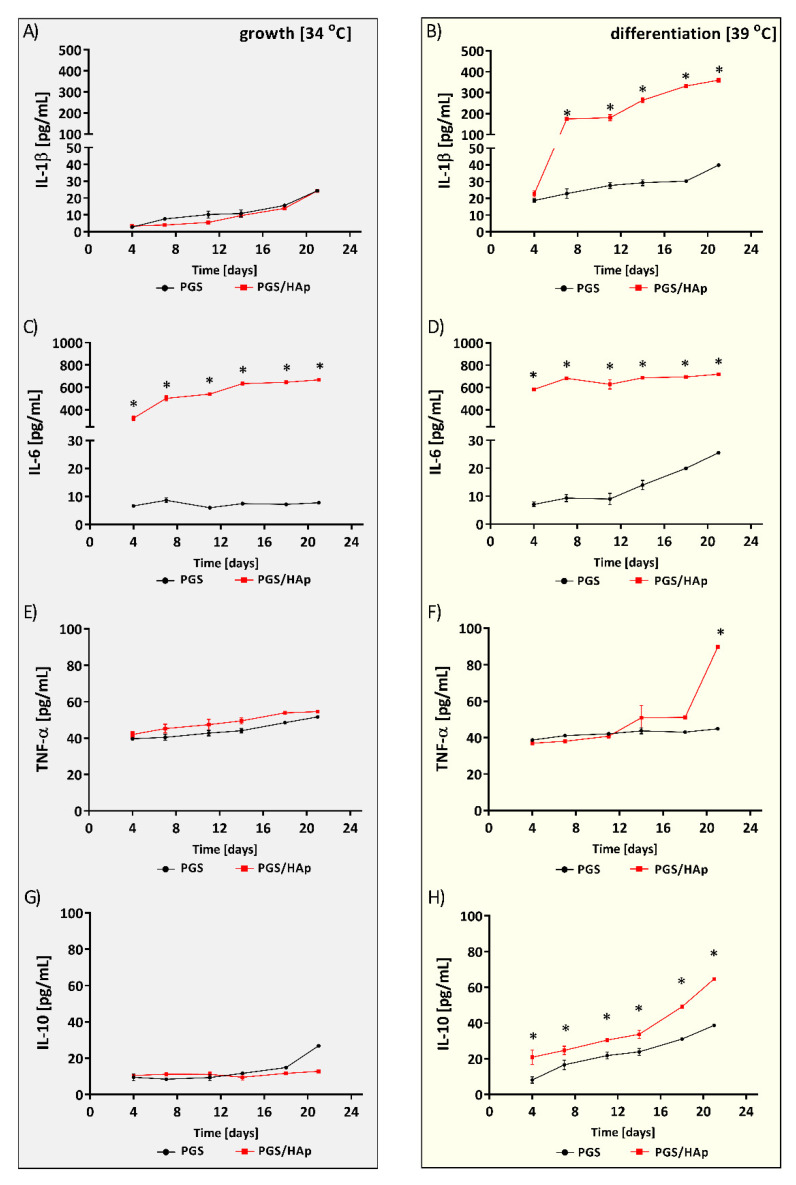
The concentrations of immunomodulatory cytokines relevant to bone homeostasis (IL-1β (**A**,**B**), IL-6 (**C**,**D**), TNF-α (**E**,**F**) and IL-10 (**G**,**H**)) assayed in the supernatants of hFOB 1.19 cultured on PGS or PGS/HAp scaffolds under a permissive temperature for growth (34 °C) or differentiation (39 °C), respectively. The results represent the mean concentration ± SD. * *p* < 0.05 between the PGS and PGS/HAp composites, the Mann–Whitney U test.

Bone remodeling is controlled by molecules of the immune system, including cytokines, cytokine/chemokine receptors, signaling molecules, and transcription factors. These soluble mediators regulate cell growth and differentiation, cell survival and gene expression [57]. Osteoblasts, as well as bone stromal cells, are capable of supporting the activation and differentiation of circulating monocytes and macrophages into osteoclasts, which is critical for bone remodeling [58]. In our study, we measured the concentration of IL-1β, IL-6, IL-10 and TNF-α in supernatants collected on days 4, 7, 11, 14, 18 and 21 of hFOB 1.19 culture on the PGS and PGS/HAp scaffolds under conditions supporting growth or differentiation (Figure 12).

IL-1β, which is considered a major osteoclast activating factor, is involved in various steps of osteoclast development and influences the balance between the bone formation and bone resorption [59]. Under conditions supporting cell proliferation (34 °C), the concentration of IL-1β stayed at a similar level throughout the entire course of the experiment. Its production was not related to the composition of the scaffold. The level of IL-1β produced by hFOB 1.19 on PGS scaffold was 2.8 ± 0.6 pg/mL on day 4 and reached 24.4 ± 0.8 pg/mL on day 21. The levels of IL-1β were 3.4 ± 0.3 pg/mL on day 4 and rose to 24.3 ± 0.7 pg/mL on day 21. Interestingly, in the osteogenic environment (39 °C), the concentration of IL-1β produced by hFOB 1.19 cultured exclusively on PGS/HAp increased over time and was significantly higher as compared to IL-1β production by osteoblasts cultured on composites made of PGS alone. For comparison, it was 22.7 ± 3.3 pg/mL and 18.7 ± 1.9 pg/mL, *p* = 0.04 on day 4 and reached the level of 359.7 ± 12.4 pg/mL and 39.8 ± 0.7 pg/mL, *p* = 0.02 on day 21 (Figure 12B). Thus, the production of IL-1b by the osteoblasts was strongly correlated with the ongoing terminal differentiation of hFOB1.19. 

Among other osteoclastogenic cytokines, the proinflammatory IL-6 is a major player contributing to the differentiation of osteoclasts and the modulation of bone resorption [60]. Interestingly, we found that the production of this cytokine by hFOB 1.19 was associated with the enrichment of the PGS scaffolds with HAp, irrespective of the culturing temperature. The concentration of IL-6 secreted by osteogenic cultured on the PGS/HAp composites at 34 °C was significantly higher at all time points, e.g., 326.4 ± 31.7 pg/mL on day 4, reaching the level of 667.9 ± 14.9 pg/mL on day 21 as compared to the IL-6 production evaluated in the supernatants collected from hFOB 1.19 cultured on neat PGS scaffolds: 6.7 ± 0.3 pg/mL (*p* = 0.02) on day 4 and 7.8 ± 0.2 pg/mL (*p* = 0.02) on day 21. The same correlation was observed in cell cultures grown under osteoinductive conditions (39 °C). Similarly to the dataset collected at 34 °C, hFOB 1.19 cultured on the PGS/HAp composites produced significantly more IL-6 as compared to cytokine production by osteoblasts on the PGS composites. The levels recorded were 582.9 ± 14.0 pg/mL and 7.1 ± 1.5 pg/mL (*p* = 0.02) on day 4 and 720.2 ± 1.3 pg/mL and 25.5 ± 0.7 pg/mL (*p* = 0.02) on day 21 (Figure 12C,D).

TNF-α is considered a potent inducer of bone resorption and an important promoter of osteoclastogenesis. As a pleiotropic cytokine, it exerts proinflammatory activities and induces osteoclast formation from bone marrow-derived macrophages. TNF-α initiates osteoclast differentiation and activates osteoclasts to resorb bone, which may be beneficial to the repair process during bone healing [61,62]. We found that the production of TNF-α was not induced in cells cultured at 34 °C, and the composition of the scaffold had no effect on the cytokine production under such conditions. The levels of TNF-α produced by hFOB 1.19 seeded on the PGS and PGS/HAp composites were 39.7 ± 1.4 pg/mL and 42.1 ± 2.7 pg/mL on day 4 and 51.7 ± 0.6 pg/mL and 54.6 ± 0.3 pg/mL on day 21. Similar observations were made when assessing the concentration of TNF-α produced by cells cultured in the osteogenic environment (39 °C). The levels were 38.7 ± 1.4 pg/mL and 36.9 ± 1.6 pg/mL on day 4 and 43.0 ± 0.2 pg/mL and 51.2 ± 0.7 pg/mL on day 18. Interestingly, on day 21, we observed a significantly higher production of TNF-α by osteoblast cultured on the PGS/HAp composite as compared to the PGS scaffold at 89.7 ± 1.0 pg/mL and 44.9 ± 0.9 pg/mL (*p* = 0.02), respectively (Figure 12E,F).

The anti-inflammatory properties of IL-10 have been implicated in bone homeostasis. This cytokine downregulates the synthesis of IL-1, IL-6, TNF-α, gelatinase and collagenase and directly inhibits osteoclast formation [63]. Under osteogenic conditions, the PGS/HAp scaffold favored the IL-10 production by hFOB 1.19 as compared to cytokines secreted by cells cultured on neat PGS at all time points, e.g., 20.9 ± 7.1 pg/mL and 8.1 ± 3.0 pg/mL, *p* = 0.02 on day 4 and 64.7 ± 0.7 pg/mL and 38.7 ± 0.6 pg/mL, *p* = 0.01 on day 21. 

In contrast, cells cultured at 34 °C produced less IL-10, and the concentration of this cytokine remained similar throughout the entire experiment. While the trend in production of IL-10 was generally not related to the composition of the scaffold, the PGS/HAp scaffolds stimulated higher IL-10 production on day 21. The level of IL-10 produced by hFOB 1.19 on the PGS scaffold was 9.6 ± 3.3 pg/mL on day 4 and reached 26.8 ± 1.0 pg/mL on day 21. In the case of PGS/HAp composites, it was 10.6 ± 0.5 pg/mL on day 4 and 12.8 ± 0.5 pg/mL on day 21 (Figure 12G,H).

### 2.6. In Vivo Biocompatibility Study

**Figure 13:**
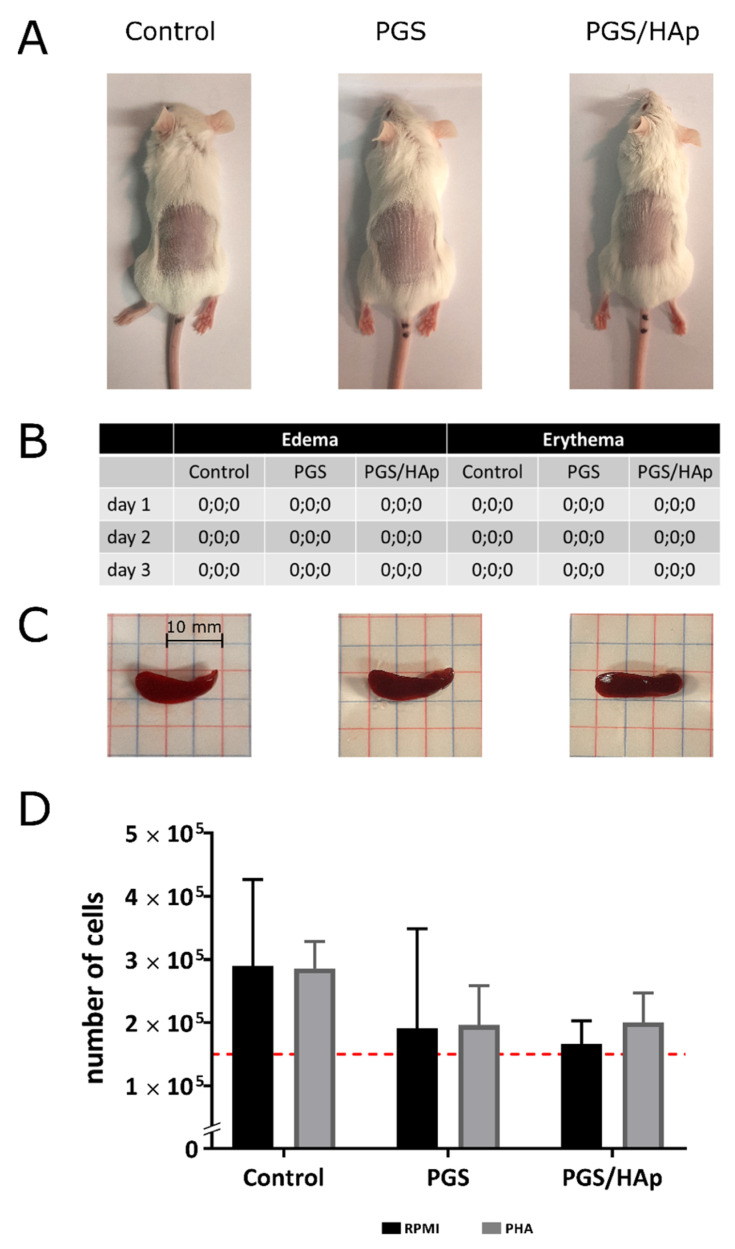
Biocompatibility testing of nonpolar extracts obtained from the PGS and PGS/HAp scaffolds. BALB/c mice were monitored following subcutaneous injections of PGS and PGS/HAp extracts or physiological saline for 72 h to screen for adverse reactions (**A**). The appearance of edema and erythema was assessed and graded daily according to the Primary Irritation Index (PII) = 0/3, where 0 indicates that the irritation is negligible (**B**). At 72 h post-injection, the animals were terminated and spleens (**C**) along with blood, samples and other critical organs were collected and inspected. Splenic lymphocytes were next isolated, and their proliferation rates were measured in the presence or absence of a mitogen—PHA (**D**) with a CyQUANT assay.

When characterizing a novel biomaterial with potential for future medical applications, it is crucially important to progress toward biocompatibility tests in vivo. In this work, initial biocompatibility tests were performed with the use of nonpolar extracts of PGS and PGS/HAp biomaterials on BALB/c mice. Following the subcutaneous injection of extracts, no signs of skin irritation were noted, judging by the complete absence of any signs of edema or erythema formation in the vicinity of the site of injection over the course of the experiment. Upon termination of animals, the internal organs looked healthy, and there were no obvious signs of inflammation. Lymphocytes isolated from the fresh spleens of the animals did not exhibit increased rates of proliferation in comparison to the vehicle control, and their proliferation was not significantly co-stimulated by the addition of PHA. All these observations suggest that the PGS and PGS/HAp scaffolds can be considered safe for future implantation trials. The results of biocompatibility tests are summarized in Figure 13.

## 3. Materials and Methods

### 3.1. Materials

In this research, sebacic acid (CAS no.111-20-6), deuterated acetone (99.9 atom % D, contains 0.03 % (*v/v*) TMS) (CAS no. 666-52-4) and Glycerol (CAS no. 56-81-5) from Aldrich^®^, NaCl from P.P.H “STANLAB” Sp.J. (CAS no. 7647-14-5) with 400–500 µm grain size and tetrahydrofuran (CAS no. 109-99-9) from POCH S.A. were used. For the synthesis of hydroxyapatite, ammonia water (25%) from “STANLAB” Sp.J. (CAS no. 1336-21-6), calcium acetate monohydrate (CAS no. 5743-23-0) and disodium hydrogen phosphate (CAS no. 755-79-4) from CHEMPUR were used.

### 3.2. pPGS Synthesis

PGS was synthesized on a Mettler Toledo EasyMax^®^ 102 chemical reactor equipped with a mechanical stirrer. The reaction was conducted without solvent with a 1:1 molar ratio of sebacic acid to glycerol. For 10 g of glycerol (0.1086 mol), an equimolar amount of sebacic acid was used (21.95 g)

**Figure 14:**
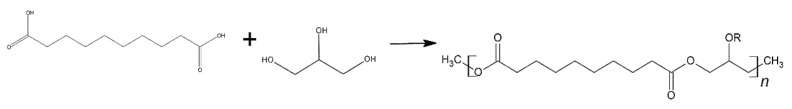
Schematic of PGS synthesis.

Sebacic acid was heated with glycerol to 130 °C and stirred mechanically. After the monomers were combined, the reaction was continued for 24 h and quenched by lowering the temperature to 25 °C, and its product was stored for further use. The reaction scheme is shown in Figure 14.

### 3.3. Hydroxyapatite Synthesis

A wet precipitation method was chosen to synthesize nano-hydroxyapatite (HAp). For this purpose, several drops of a 25% ammonia water were added to a solution of Na_2_HPO_4_ (80 mL) and H_2_O (520 mL) to obtain an alkaline environment (pH > 10). The mixture was then heated to its boiling point and, when the boiling temperature was reached, a solution of hydrated Ca(CH_3_COO)_2_·H_2_O (200 mL) was added dropwise under constant stirring at a rate of 1 drop/s. After the reaction was completed, the obtained suspension with the precipitate was left for 24 h to set the HAp crystals at room temperature. Next, the precipitate was washed thoroughly with distilled water to remove any excess ammonia water and unreacted substrates. The washed product was then centrifuged in a rotary centrifuge (MPW-233E). The supernatant was decanted, and the obtained HAp was freeze-dried.

### 3.4. PGS and PGS/HAp Porous Scaffold Preparation (TIPS-TCL-SL Process)

The microporous foams were produced by preparing a series of a 20 wt.% solution of pPGS in 1,4-dioxane and dissolving it on a magnetic stirrer over 24 h. For the composites with HAp, a hydroxyapatite was added to the mixture with a 75/25 PGS/HAp mass ratio. The HAp was stirred in the solution for another 24 h, and the sample was sonicated three times for 15 min every few hours and one additional time before transferring the solution to multi-well plates. 

Next, in a Teflon 24-well plate, 1.5 g of 400–500 µm NaCl was added to each well, and 0.75 mL of the solution was poured on top. The samples were frozen for 24 h and freeze-dried for another 24 h afterwards. Then, the composites were cured in the plates at 185 °C for 2.5 h. Finally, the salt was leached from the foams using a 10 L beaker with distilled water. The salt leaching process lasted 24 h, and the water was changed three times before being stirred overnight. The foams were dried at 40 °C for 24 h. Our sample composition is described in Table 4.

### 3.5. Physicochemical Properties of the Scaffolds

#### 3.5.1. Chemical Structure Identification (NMR)

Proton nuclear magnetic resonance (^1^H NMR) was performed on a Avance 400 MHz spectrometer (Bruker, Billerica, United States) operating at 20 °C, with a 3.3 s acquisition time, a 5.0 s relaxation delay, a spectra width of 8013 Hz, 32 scans, 32,769 points and a free induction decay (FID) resolution of 0.3 Hz. All the samples for the ^1^H NMR analysis were dissolved in deuterated acetone. All the details and calculations are given in the Supporting Info file.

#### 3.5.2. Attenuated Total Reflectance-Fourier Transform Infrared Spectroscopy

The Attenuated Total Reflectance-Fourier Transform Infrared Reflectance spectra (ATR-FTIR) were recorded with a Nicolet iZ10 spectrometer (Thermo Scientific, Waltham, MA, USA) equipped with a Smart iTR™ diamond ATR accessory. The spectra were acquired with a resolution of 4 cm^−1^ in the range of 4000–550 cm^−1^ (32 co-added scans).

#### 3.5.3. Gel Permeation Chromatography (GPC)

The pPGS sample was dissolved in tetrahydrofuran (THF), and the GPC was performed on a Waters Aliance 2695 chromatograph equipped with three 7.8 × 300 mm columns and a Waters 1414 refractive index detector. The measurement was held at the THF flow rate at a level of 0.6 mL/min. The calibration curve was based on the polystyrene standards in the range of 1.31 × 103 to 3.64 × 106 Da.

#### 3.5.4. Thermogravimetry (TGA)

The TGA measurements were performed utilizing a TGA/DSC1 Mettler Toledo system. Samples were heated 10 °C/min in the temperature range of 25 to 900 °C under 60 cm^3^/min of nitrogen flow.

#### 3.5.5. Differential Scanning Calorimetry (DSC)

The DSC curves were registered using a DSC1 STARe Differential Scanning Calorimeter System from Mettler-Toledo. The measurements were carried out in the temperature range of −70 to 250 °C with a heating/cooling rate of 10 K/min. The samples were measured in aluminum pans under a constant nitrogen purge (60 mL/min). First, cooling and, second heating thermographs were used for the analysis.

#### 3.5.6. Scaffold Micro-Imaging

To evaluate the complex morphology of scaffold cross-sections on different scales and levels, the PGS and PGS/HAp porous scaffolds were evaluated using both scanning electron microscopy (SEM) (Phenom ProX; Thermo Scientific, Waltham, MA, USA) and stereoscopic microscopy (Stereo Discovery V20, Zeiss, Jena, Germany). The cross-sections of samples were prepared on a cryostat (Hyrax C50, Zeiss, Jena, Germany) at −25 °C. The diameters of pores present on the SEM microphotographs were measured 10 times at different angles in the ImageJ software. The presented value is the number-average of all the measurements.

#### 3.5.7. Microtomography (µCT)

To examine the microstructural properties, foam specimens were scanned using microtomography (SkyScan 1172, Bruker, Belgium). The samples were scanned using no filter, with a resolution of 9 µm and a 180-degree scanning range. An X-ray lamp intensity of 222 µA, voltage energy of 44 kV, rotation step of 0.25° and exposure time of 500 ms were used. After the image reconstruction (NRecon, Bruker, Belgium), cylindrical volumes of interest (VOIs) were created within each foam. The created VOIs had a diameter of 10 mm and a height of 3.5 mm. An analysis of microstructural parameters (CtAn, Bruker, Belgium) was conducted using three-dimensional parameters describing the foam porosity including the total porosity (Po(tot) [%]), open porosity (Po(op) [%]), closed porosity (Po(cl) [%]), open and closed pore volume (Po.V(op), Po.V(cl) [mm^3^]) pore size (volume-equivalent sphere diameter, ESDv [µm]), major pore diameter (Maj.Dm [µm]) and sphericity (Sph [-]).

### 3.6. Sterilization of the PGS and PGS/HAp Scaffolds

Prior to the biological evaluation, the scaffolds were sterilized by gamma radiation (35 kGy; 60Co source) at the Institute of Applied Radiation Chemistry at Lodz University of Technology (Lodz, Poland).

### 3.7. Biological Properties of the Scaffolds

**Figure 15:**
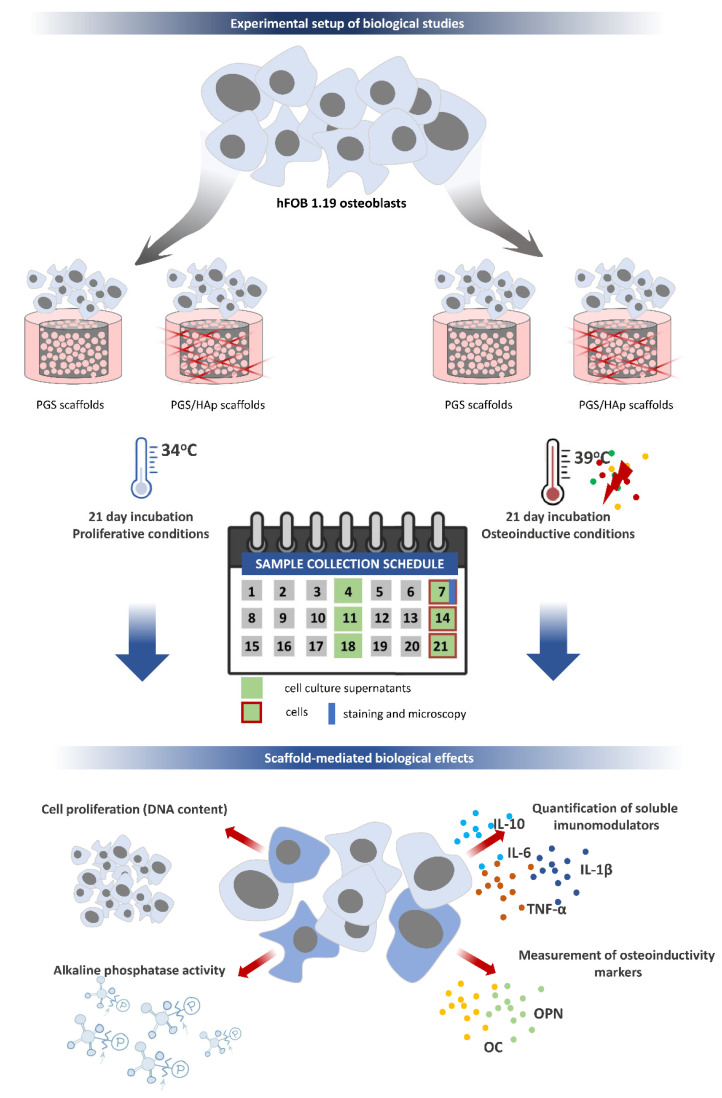
Experimental set up of the biological studies. Schematic representation of the experimental design for testing the scaffold-mediated biological effects toward pre and differentiated human osteoblasts hFOB1.19. The timeline for the assessment of cell proliferation, quantification of differentiation and immunomodulation markers is presented.

PGS and PGS/HAp scaffolds were subjected to evaluation of their biological properties. The summary of experimental design regarding scaffold-mediated biological effects (hFOB 1.19 cell proliferation, quantification of differentiation and the immunomodulatory marker levels) is presented in Figure 15.

#### 3.7.1. Cell Culture

The human fetal osteoblastic cell line hFOB 1.19 (CRL-11372™) was obtained from the American Type Culture Collection (ATCC, Manassas, VA, USA). The cells were cultured in Dulbecco’s Modified Eagle’s Medium/Ham’s Nutrient Mixture F12 (1:1 DMEM/F12 Modified; Gibco; Thermo Fisher Scientific, Waltham, MA, USA) containing a 0.3 mg/mL geneticin (Sigma-Aldrich, Saint Louis, MO, USA) and supplemented with a 10% fetal bovine serum (HyClone, Laboratories Inc., Marlborough, MA, USA). 

The cell cultures were incubated at 34 °C in a humidified air atmosphere containing 5% of CO_2_ (CO_2_ incubator; Nuaire, Plymouth, MN, USA). To ensure that the cells formed confluent and homogeneous monolayers, the culture growth and fitness were monitored using an inverted microscope (Motic AE2000 Xiamen, China). The medium was changed every 3 days under aseptic conditions. Passaging of confluent cell monolayers was carried out at 80–90% confluence using a 0.5% trypsin-0.05 mM EDTA solution (Gibco, Thermo Fisher Scientific, Waltham, MA, USA). The cell density and viability were assessed using a hemocytometer and trypan blue exclusion assay, respectively.

#### 3.7.2. hFOB 1.19 Osteoblast Growth and Differentiation

Foam scaffolds were placed into 24-well culture plates (Nunclon Sphera, Nunc, Thermo Fisher Scientific, Waltham, MA, USA) and stabilized with a tissue adhesive (SuperVet Glue, EVi-MED, Liszki, Poland). The biomaterials were incubated at 34 °C and 5% of CO_2_ for 24 h with 1 mL of hFOB 1.19 medium. Following the initial soaking, the medium was aspirated, and 20 µLs of fresh cell suspensions (2 × 10^5^ cells) were spotted at the center of each scaffold. Next, the cells were incubated for 2 h in a humidified 5% CO_2_ atmosphere at 34 °C, and then the wells were filled with 1 mL of fresh medium. The cells seeded onto scaffolds were incubated at 34 °C to support cell proliferation. 

To visualize immature osteoblasts within PGSs and PGS/HAp, the scaffolds were preincubated with cells for 7 days, washed with PBS and fixed with a 3.7% formaldehyde (Sigma-Aldrich, Saint Louis, MO, USA) solution for 10 min. The samples were mounted in a Frozen Section Media 22 (Leica Biosystems Inc., Buffalo Grove, IL, USA) and sectioned on a cryostat (Leica CM1950, Leica Biosystems Inc., Buffalo Grove, IL, USA). The resulting 20 µm-thick individual sections were stained using the May–Grunwald–Giemsa protocol and analyzed under a light microscope (Nikon ECLIPSE 50i, Nikon Inc., Tokyo, Japan) at the Laboratory of Microscopic Imaging and Specialized Biological Techniques at the University of Lodz.

For parallel experiments, the composites with hFOBs 1.19 were cultured at 39 °C in osteogenic medium, additionally supplemented with 50 μg/mL of ascorbate-2-phosphate (Sigma-Aldrich, Saint Louis, MO, USA), 1 μM of dexamethasone (Sigma-Aldrich, Saint Louis, MO, USA) and 10 mM of β-glycerophosphate (Sigma-Aldrich, Saint Louis, MO, USA).

For both culture conditions, the medium was replaced every three days. Cell culture supernatants were collected after 4, 7, 11, 14, 18 and 21 days of incubation and stored at −80 °C until further evaluation of the soluble markers of osteogenic differentiation. 

#### 3.7.3. Cell Proliferation Assay

To assess the cell proliferation within the foam scaffolds, cells cultured under osteogenic conditions were washed with PBS and frozen at −20  °C at selected time points. The cellular DNA content was quantified using a CyQUANT^®^ Cell Proliferation Assay (Invitrogen, Thermo Fisher Scientific, Waltham, MA, USA). The samples were thawed at room temperature and the cells were lysed in a buffer containing the CyQuant-GR dye, which binds to cellular nucleic acids. Fluorescence was measured at an emission wavelength of 520 nm and excitation wavelength of 480 nm using a SpectraMax^®^ i3x Multi-Mode Microplate Reader (Molecular Devices, San Jose, CA, USA).

#### 3.7.4. Quantification of Osteocalcin and Osteopontin

The osteocalcin (OC) and osteopontin (OPN) levels were determined in culture supernatants collected on day 4, 7, 11, 14, 18 and 21 of the hFOB 1.19 culture on the PGS and PGS/HAp scaffolds using enzyme-linked immunosorbent assays (ELISA). The quantitative determination of OC and OPN was performed according to the manufacturers’ instructions (Wuhan Fine Biotech Co., Wuhan, China) and normalized against appropriate standard curves. In brief, wells on the 96-well plate pre-coated with capture antibody (against OC or OPN) were washed two times with wash buffer and 100 µL of standard (OC: 1.25–80 ng/mL; OP: 0.156–10 ng/mL), samples (diluted 2× with sample dilution buffer) or controls (blank) were added to each well and incubated for 90 min at 37 °C. Next, the wells were washed again and the biotin-labeled antibody working solution (detection antibody against OC or OPN, diluted 1:100) was added to each well (100 µL) and incubated for 60 min at 37 °C. Following washing, streptavidine-horseradish peroxidase (SaV-HRP)-solution (diluted 1:100) was added and incubated for 30 min at 37 °C. The tetramethylbenzidine (TMB) substrate was used to visualize the HRP enzymatic reaction. The colorimetric reaction was stopped within 20–30 min, and the absorbance values were determined at 450 nm using a Multiskan EX reader (Thermo Scientific, Waltham, MA, USA).

#### 3.7.5. Alkaline Phosphatase Activity

The alkaline phosphatase activity (ALP) was measured in samples used for the cell DNA content evaluation in a p-NPP (para-nitrophenylphosphate) hydrolysis assay. We added 100 µL of p-NPP (at a rate of 4 µg/µL) to 100 µL of cell lysate samples on a 96-well plate and incubated at 37 °C for 30 min. Then, the stop solution of 2 M NaOH was added to stop the enzymatic reaction. The optical density at 405 nm was recorded for each sample using a Multiskan EX reader (Thermo Scientific, Waltham, MA, USA). The standard curve (ranging from 0 to 10 U/mL) of ALP (Molecular Biology, Thermo Scientific, Waltham, MA, USA) was used to calculate the resulting enzymatic activity in each cell lysate.

#### 3.7.6. Determination of the Cytokine Release Profile

The concentrations of IL-1β, IL-6, IL-10 and TNF-α in the culture media of hFOB 1.19 cells incubated with PGS and PGS/HAp scaffolds collected on days 4, 7, 11, 14, 18 and 21 were assessed by ELISA (BD OptEIA™ Set, BD-Pharmingen, Franklin Lakes, USA) according to the manufacturer’s instructions. In brief, wells of a 96-well half area plate (Greiner Bio-One, Greiner Bio-One GmbH, Kremsmünster, Austria) were coated with capture antibodies in 0.1 M sodium carbonate, incubated overnight at 4 °C, then washed with PBS/0.05% Tween 20 and blocked with PBS/10% FBS for 1 h. Following subsequent washing, the samples were added and incubated for 2 h. Standard curves were established using serial dilutions of known concentrations of recombinant human cytokines (IL-1β: 250-3.9 pg/mL, IL-6: 300-4.7 pg/mL, IL-10: 500-7.8 pg/mL and TNF-α: 500-7.8 pg/mL). Next, the wells were washed with PBS/0.05% Tween 20 again and incubated with biotinylated monoclonal antibody in PBS/10% FBS for 1 h. Next, the wells were washed with PBS/0.05% Tween 20 for the third time and incubated with SAv-HRP in PBS/10% FBS for 30 min. After washing the wells with PBS/0.05% Tween 20, a mixture of TMB and hydrogen peroxide was added. The colorimetric reaction was stopped with 2 N H2SO4. The absorbance values were determined at 450 nm (with a correction at 570 nm) using a Multiskan EX reader (Thermo Scientific, Waltham, MA, USA). The limit of detection was 3.9 pg/mL for IL-1β, 4.7 pg/mL for IL-6 and 7.8 pg/mL for IL-10 and TNF-α, respectively.

#### 3.7.7. Determination of the Cytokine Release Profile

The concentrations of IL-1β, IL-6, IL-10 and TNF-α in the culture media of hFOB 1.19 cells incubated with PGS and PGS/HAp scaffolds collected on days 4, 7, 11, 14, 18 and 21 were assessed by ELISA (BD OptEIA™ Set, BD-Pharmingen, USA) according to the manufacturer’s instructions. In brief, wells of a 96-well half area plate (Greiner Bio-One, Greiner Bio-One GmbH, Kremsmünster, Austria) were coated with capture antibodies in 0.1 M sodium carbonate, incubated overnight at 4 °C, then washed with PBS/0.05% Tween 20 and blocked with PBS/10% FBS for 1 h. Following subsequent washing, the samples were added and incubated for 2 h. Standard curves were established using serial dilutions of known concentrations of recombinant human cytokines (IL-1β: 250-3.9 pg/mL, IL-6: 300-4.7 pg/mL, IL-10: 500-7.8 pg/mL and TNF-α: 500-7.8 pg/mL). Next, the wells were washed with PBS/0.05% Tween 20 again and incubated with biotinylated monoclonal antibody in PBS/10% FBS for 1 h. Next, the wells were washed with PBS/0.05% Tween 20 for the third time and incubated with SAv-HRP in PBS/10% FBS for 30 min. After washing the wells with PBS/0.05% Tween 20, a mixture of TMB and hydrogen peroxide was added. The colorimetric reaction was stopped with 2 N H_2_SO_4_. The absorbance values were determined at 450 nm (with a correction at 570 nm) using a Multiskan EX reader (Thermo Scientific, Waltham, MA, USA). The limit of detection was 3.9 pg/mL for IL-1β, 4.7 pg/mL for IL-6 and 7.8 pg/mL for IL-10 and TNF-α.

#### 3.7.8. In Vivo Biocompatibility Assessment

All studies involving animals were approved by the Local Ethics Committee (LKE9) for Animal Experiments of the Medical University of Lodz, Poland, which was established by the Ministry of Science and Higher Education in Poland (Decision 7/ŁB192/2021). Both genders of BALB/c adult mice (20–25 g), free of pathogens were bred and housed in the Animal House at the Faculty of Biology and Environmental Protection, University of Lodz (Poland). The animals were kept in air-conditioned rooms at 20–24 °C in cages with free access to drinking water and food pellets ad libitum. They were exposed to a 12 h light/dark cycle. Approximately 24 h before the test, the animals were shaved from the dorsal area at the central site of the trunk, then subcutaneously administered 0.2 mL of the tested extracts or vehicle control (three animals per group were done). Nonpolar extracts of biomaterials were prepared according to the norm PN EN ISO 10993-12:2012E. Briefly, tested composites (irregularly shaped porous materials): PGS and PGS modified with Hydroxyapatite (PGS/HAp) were incubated with physiological saline for 72 h at 37 °C (the extraction ratio of surface area or mass to volume was 0.1 g/1 mL). The animals were monitored daily (water and food intake as well as behavioral symptoms) and skin reactions, defined as erythema and edema, were evaluated after 24, 48 and 72 h according to a skin reaction scoring system [64]. After 72 h, the animals were euthanized with an overdose of sodium barbiturate (Morbital, Biowet, Pulawy, Poland), and then the blood and organs (spleen, liver, and lymph nodes) were collected and examined for any signs of pathologies. The activation of splenic lymphocytes was next assessed by measuring their proliferation rate with and without the addition of phytohemagglutinin (PHA) (Sigma-Aldrich, Saint Louis, MO, USA). The total spleen lymphocytes were isolated according to a previously published protocol [65]. Briefly, splenic cell suspensions were prepared in RPMI 1640 (Sigma-Aldrich, Saint Louis, MO, USA) supplemented with 10% fetal bovine serum (FBS), 2 mM L-glutamine (Sigma-Aldrich, Saint Louis, MO, USA) and 100 μg/mL penicillin/streptomycin (Sigma-Aldrich, Saint Louis, MO, USA). The cell density was assessed using a hemocytometer, and the cells were plated on a 96-well plate at a density of 2.5 × 104 cells/well. When appropriate, PHA was then added to the final concentration of 5 µg/mL. Following 72 h cultivation, the culture medium was removed, and cells were frozen at −70 °C until further processing. Proliferation assessment was performed with the use of CyQUANT reagent as described in earlier sections of the manuscript.

#### 3.7.9. Statistical Analysis

The statistical analysis was performed using GraphPad Prism 6 (GraphPad Software, San Diego, CA, USA). The Kolmogorov–Smirnov test was used to test the normality of the data. For the variables that did not present normal distribution, the Mann–Whitney U test was used for comparison between both groups. All the experiments were carried out in triplicate, and *p* < 0.05 was considered as statistically significant.

## 4. Conclusions

In our research, we successfully prepared a PGS/HAp (75/25 wt. ratio) scaffold using thermally induced phase separation supported by thermal crosslinking and salt leaching. The PGS/HAp scaffold porosity was ~70% and the pore size was ~400 µm (Maj.Dm). The water contact angle of the foams was lower for the PGS/HAp composite (~112°) than for the neat PGS (~119°). Our DSC experiments proved that the PGS/HAp composite was amorphous, whereas pPGS, and PGS was semicrystalline with a lower crystallinity degree for the PGS foam.

Due to their biodegradability, good biocompatibility and mechanical properties, the potential utility of PGS-based biomaterials for bone tissue engineering and regeneration applications has been attracting increasing attention. We are the first to show that the osteoblastic cells hFOB 1.19 readily inhabit and proliferate on such scaffolds, which indicates that there is no cytotoxic effect. It is worth emphasizing that the addition of HAp to the PGS scaffold displayed osteoconductive properties as evidenced by the production of osteoblast differentiation factors. Importantly, the cells cultured on PGS/HAp composites secreted cytokines critical for coordination of osteoclastogenesis, thus, increasing the chances of reabsorption of the composite and coordinated bone remodeling. Our initial evaluation of the biocompatibility of PGS-base composites in vivo revealed that the scaffolds should not induce any severe adverse reactions in animal models, and future studies may safely expand toward implantation trials.

Overall, the biological assessment of the PGS/HAp foams places them as a very promising candidate suitable for future biomedical engineering applications.

## Figures and Tables

**Table 1:** The relative density and porosity of the scaffolds and the substrates.

Parameter	PGS	PSG/HAp
Po(tot) ^a^ [%]	78	69.8
Po(op) ^a^ [%]	78	69.6
Po(cl) ^a^ [%]	>0.001	0.02
Po.V ^a^ [mm^3^]	216.01	193.39
ESDv [µm]	383 ± 120	286 ± 103 304 ± 77 *
Maj.Dm [µm]	596 ± 271	385 ± 147 410 ± 115 *
Sph [-]	0.81 ± 0.05	0.72 ± 0.12

^a^ Parameters obtained from the 3D analysis; * values for foam with excluded micropores observed within walls during analysis; results shown as the mean value ± SD.

**Table 2:** Thermal stability set as temperature of 5 wt.%”. loss (T_-5%_) and mass loss at 700 °C of hydroxyapatite, prepolymer and porous scaffolds PGS100 and PGS/HAp 75/25.

Sample	T_-5%_ [°C]	Mass Loss at 700 °C [%]
HAp	513	5.79
pPGS	305	76.99
PGS	360	97.02
PGS/HAp	367	98.47

**Table 3:** The thermal parameters of pPGS, PGS100 and PGS/HAp 75/25 evaluated from the cooling and second heating DSC curves.

Sample	Cooling			2nd Heating		
	T_g_ [°C]	T_c_ [°C]	ΔH_c_ [J/g]	T_g_ [°C]	T_m_ [°C]	-ΔH_c_ [J/g]
pPGS	-	−17.8	18.5	−25.1	3.3	18.6
PGS	-	−23.6	7.7	−25.8	−9.4	3.7
PGS/HAp	−28.6	-	-	−24.9	-	-

**Table 4:** Sample designations.

	PGS Content [wt.%]	PGS Content [wt.%]
PGS	100	-
PGS/HAp	75	25

## Data Availability

Data is contained within the article or supplementary material.

## Supplementary Materials

The following are available online at https://www.mdpi.com/article/10.3390/ijms22168587/s1.
