# Supplementary material for: PGS/HAp Microporous Composite Scaffold Obtained in the TIPS-TCL-SL Method: An Innovation for Bone Tissue Engineering
Source: Int J Mol Sci. 2021 Aug 10;22(16):8587. doi: 10.3390/ijms22168587 (PMC8395318; doi:10.3390/ijms22168587)
Supplement: Supplementary file 1 [file ijms-22-08587-s001.zip › ijms-1321049-supplementary.pdf]

Supporting Information

Osteoinductivity of PGS/HAp microporous composite scaffold obtained in the TIPS-TCL-SL method: an innovation for bone tissue engineering

1. Experimental section

NMR data

Molar fraction of an unreacted glycerol was calculated according to the following equation (**equation S1**):

$$x_{UG} = \frac{I_{3.44-3.84ppm} - [2I_{(5)} + 4I_{(4)} + 2I_{(2)}]}{5} \text{ (Equation S1)}$$

where $I_{3.44\text{-}3.84ppm} = I_{(4'')} + I_{(5')} + I_{(2'')} + I_{(UG)}$ is the area under the signal in the range from 3.44 to 3.84 ppm; $I_{(5)}$ is the integral of the signal related to the methine proton in the terminal T2 glycideric unit, $I_{(4)}$ is the integral of the signal related to the methine proton in the terminal T1 glycideric unit and $I_{(2)}$ is the integral of the signal related to the methine proton in L12 diglycideric subunit.

The molar fraction of the L13 glyceridic unit was determined according to the following equation (**equation S2**):

$$x_{L13} = 1 - [x_{L12} + x_D + x_{T1} + x_{T2} + x_{UG}] \text{ (Equation S2)}$$

where x_i is the molar fraction of the glyceridic unit type[1].

Carboxylic acid conversion (p_{COOH}) was estimated by Lorentzian fitting of the overlapped signal *a* to derive the relative areas of component signals *a'* and *a''*, related to the monoester- and diester type of sebacate subunit. Then the conversion of carboxyl groups was calculated according to the following equation (**equation S3**):

$$p_{COOH} = \frac{I_{a''}}{I_{a'} + I_{a''}} \text{ (Equation S3)}$$

where $I_{a'}$ and $I_{a''}$ are the integrals of the monoester- and diester-type of sebacate unit, respectively. The degree of substitution (DS), hydroxyl conversion (p_{OH}), number average degree of polymerization (DP) and degree of branching (DB) were calculated according to the methodology reported elsewhere[1,2]. All of the parameters describing pPGS prepolymer derived from ^{1}H NMR spectrum are presented in **Table S1**

Water contact angle measurement

Water contact angle measurements were conducted with a PG-X contact angle goniometer (Testing Machines, Inc.). Nine measurements were carried out for each specimen. The mean value was calculated as the standard deviation.

2. Results

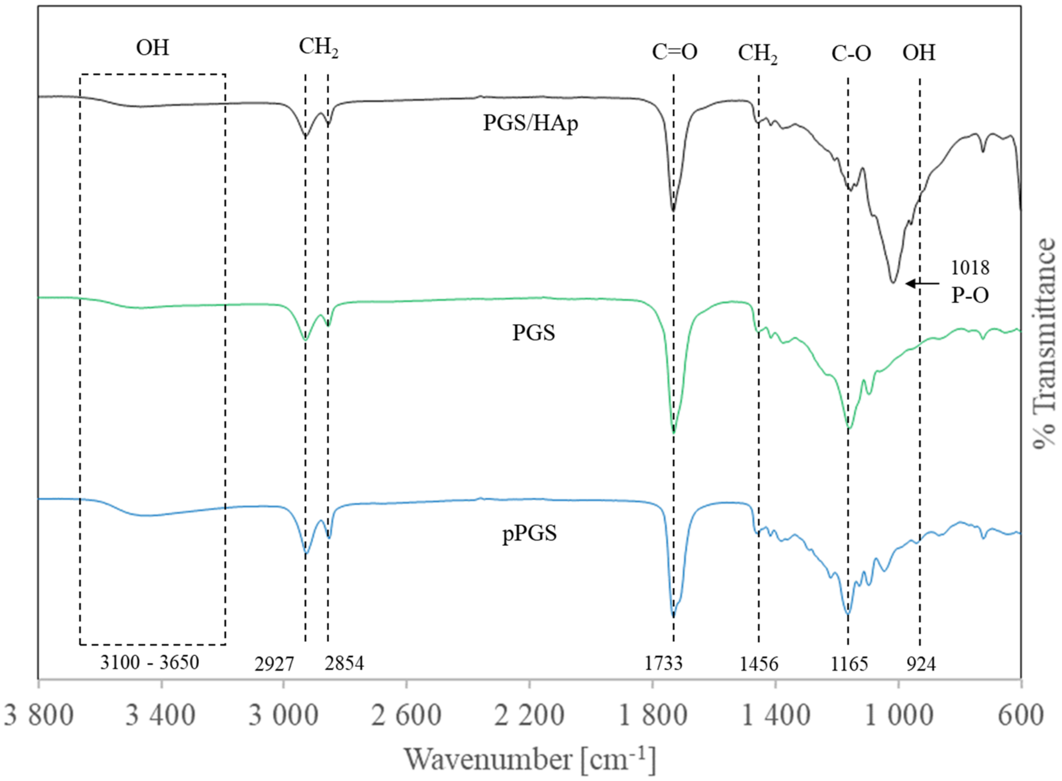

Figure S1. ATR-FTIR spectra of pPGS, PGS and PGS/Hap

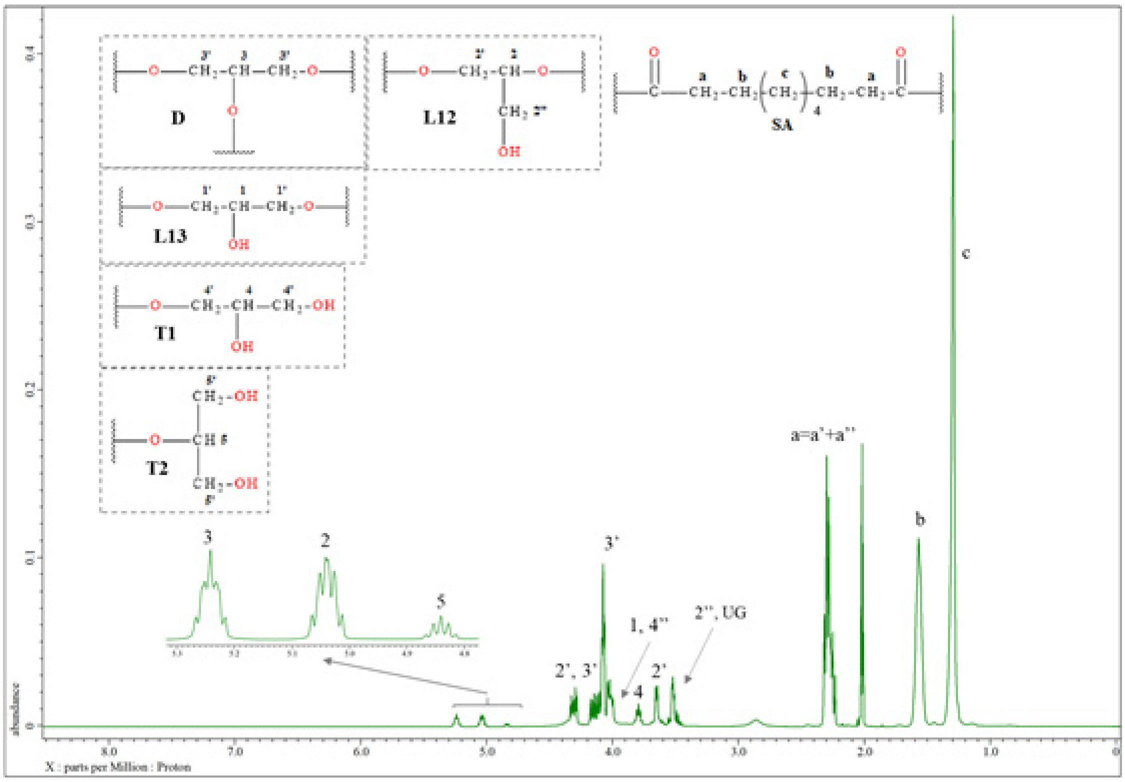

Figure S2. ^{1}H NMR spectrum in $(CD_3)_2CO$ of pPGS prepolymer with signals assignment (T1, T2 – terminal glyceridic units, L12, L13 – diglyceridic unit, D – triglyceridic unit, UG – unreacted glycerol, SA – sebacate subunit)

Table S1. Pre-polymer parametrs determined by ^{1}H NMR (x_i – average molar fraction of different types of glyceridic units in reaction medium, p_{COOH} – carboxylic acid conversion, p_{OH} – hydroxyl groups conversion, DP – average degree of polymerization, DS – degree of substitution, DB – degree of branching)

x_{UG}	x_{T1}	x_{T2}	$x_{DG1,2}$	$x_{DG1,3}$	$x_{TG1,2,3}$	p_{OH}	p_{COOH}	DP	DB	DS
**0.03**	0.24	0.03	0.16	0.38	0.16	0.63	0.91	10.2	0.37	1.89

A)

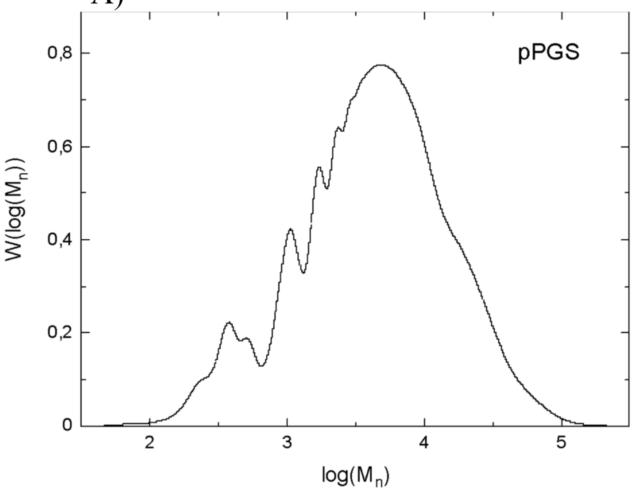

B)

M_n [Da]	M_w [Da]	M_z [Da]	PDI	Peak area [%]
6362	11867	26455	1.865	66.84
2252	2265	2277	1.005	6.71
1640	1659	1678	1.012	8.05
977	1009	1044	1.033	9.18
316	379	428	1.198	8.77

Figure S3. A) molecular weight distribution plot for pPGS, B) molecular weight distribution characterization

References

[1] G. B. Perin, M. I. Felisberti, *Macromolecules* **2020**, *53*, 7925.

[2] C. Moorhoff, Y. Li, W. D. Cook, C. Braybrook, Q.-Z. Chen, *Polym. Int.* **2015**, *64*, 668.
